# The influence of hypoxia on cancer lipid metabolism: a scoping review

**DOI:** 10.1186/s12943-026-02758-8

**Published:** 2026-08-05

**Authors:** Henry Douglas Robb, Philip Kwan Hung Leung, Haeun Kim, Daniel Campion-Norman, Bibek Das, Robert Bristow, George Bushra Hanna

**Affiliations:** 1 Department of Surgery and Cancer, Imperial College London, Hammersmith Hospital Campus, Du Cane Road, London, W12 0NN UK; 2 Imperial College Healthcare NHS Trust, London, UK; 3 Translational Oncogenomics Laboratory, Cancer Research UK Manchester Institute, University of Manchester, Manchester, UK; 4 Manchester Cancer Research Centre, University of Manchester, Manchester, UK

**Keywords:** Tumour Hypoxia, Cancer Metabolism, Lipidomics, Metabolomics, Cancer Signalling

## Abstract

**Background:**

Hypoxia, a hallmark of solid tumours, profoundly alters cellular metabolism to enable survival in the harsh tumour microenvironment. Whilst effects on carbohydrate and protein metabolism are well characterised, the hypoxic regulation of lipid metabolism remains poorly understood. Recent advances in lipidomics have revealed that lipids not only serve as energy resources, but have critical roles in oncogenic signalling, cell homeostasis and membrane metabolism. Understanding hypoxia-driven lipid reprogramming could uncover new therapeutic opportunities.

**Methods:**

A scoping review was conducted following internationally accepted guidelines and reported using the PRISMA-ScR Checklist. MEDLINE, Embase, and Web of Science were searched from inception to 31st August 2025 using tailored strategies, supplemented with grey literature and reference snowballing. Eligible studies included original peer-reviewed research investigating hypoxia and lipid metabolism in cancer using in vitro, in vivo, or human models with physiologically relevant hypoxic conditions. Data were extracted and thematically analysed across four domains: lipid accumulation, lipolysis, membrane metabolism, and lipid signalling.

**Results:**

From 4632 records identified, 53 studies met the inclusion criteria. Most investigations used immortalised 2D cancer cell lines exposed to chronic, moderate hypoxia (1–2% O₂). Thematic analysis revealed that the majority of studies focused on lipid accumulation, particularly through lipid droplet formation, *de novo* lipogenesis, and exogenous lipid uptake. Lipolysis, membrane metabolism, and lipid signalling under hypoxic conditions were less frequently examined. Together, these findings highlight a predominant emphasis on lipid storage pathways, with comparatively limited exploration of lipid degradation and signalling processes. A narrative synthesis summarised key mechanisms, identified major knowledge gaps, and outlined emerging therapeutic opportunities in hypoxia-driven lipid metabolism.

**Conclusions:**

Hypoxia drives diverse, cancer-specific lipidomic adaptations that promote survival and treatment resistance. Although limitations in current models and experimental conditions constrain translation, advancing our understanding of hypoxia-driven lipid reprogramming presents promising avenues for therapeutic development and clinical application.

**Supplementary Information:**

The online version contains supplementary material available at https://doi.org/10.1186/s12943-026-02758-8.

## Introduction

All solid tumours are dependent on angiogenesis to support their nutrient and oxygen requirements for continued growth. Invariably, the rapid cellular proliferation inherent to cancer leads to a chaotic microvascular architecture incapable of meeting the tumour’s metabolic demands [1]. Such imbalance between oxygen demand and supply, termed hypoxia, has become a hallmark of solid cancers, affecting up to 90% of tumours [2]. Across multiple malignancies, highly hypoxic tumours have been consistently associated with poorer disease-free and overall survival [3]. This is attributable to a combination of factors, including inherent resistance to oncological therapies, genomic instability and propensity to metastasis [4–7]. The impact of tumour hypoxia on clinical outcomes has made it a focus of oncological research.

In the hypoxic tumour microenvironment, as tissue pO_2_ levels drop below 10 mmHg (1–2% O_2_), adaptive survival pathways are activated with profound metabolic effects [8]. For example, it is well established that the oxygen-sensing and key transcriptional regulator hypoxia-inducible factor (HIF) reprogrammes carbohydrate metabolism by potentiating the Warburg-like phenotype, whereby cancer cells preferentially depend on glycolysis rather than mitochondrial oxidative phosphorylation [9]. Similarly, protein metabolism is significantly altered. HIF-dependent upregulation of protein Regulated in Development and DNA Damage-1 (REDD-1) suppresses Mammalian Target of Rapamycin Complex-1 (mTORC1) via the Tuberous Sclerosis Complex-1 and − 2 (TSC1/TSC2), altering protein metabolism to conserve energy under hypoxic conditions [10, 11]. Hypoxia also regulates gene expression through HIF-independent mechanisms, including direct effects on oxygen-dependent chromatin-modifying enzymes, whereby inhibition of histone demethylases can alter histone methylation and epigenetic transcriptional programmes [12, 13]. In contrast, our understanding of hypoxic tumour lipid metabolism has only recently advanced following crucial analytical technological innovations [14].

Lipids are a diverse and complex class of biomolecules, encompassing fatty acyls, glycerolipids, glycerophospholipids, sphingolipids, sterol lipids and prenol lipids. Their fundamental roles in cancer cell survival and progression as an energy source and as building blocks for membrane biosynthesis, have long been recognised [15]. However, more nuanced functions as potent signalling molecules and modulators of reactive oxygen species (ROS) have become evident [16]. Unsurprisingly, hypoxic tumours have a profoundly deranged lipidome [17]. These hypoxia-associated lipid alterations may also influence vulnerability to lipid peroxidation-mediated cell death pathways, including ferroptosis, which is shaped by membrane phospholipid composition, iron availability and cellular redox state [18–20]. Characterising these changes using innovations within cancer lipidomics offers promising avenues for identifying biomarkers, prognostication, drug discovery and therapeutic monitoring [21–23].

Here, we present a scoping review summarising current evidence on the influence of hypoxia on cancer cell lipid metabolism. Our aim is to highlight key metabolic alterations, identify gaps in the literature, and outline opportunities for future translational and clinical applications.

## Methods

An a priori scoping review protocol was developed following internationally accepted standards and guidelines [24, 25]. Findings are reported as per the Preferred Reporting Items for Systematic Review and Meta-analyses extension for Scoping Review (PRISMA-ScR) checklist [26].

### Search strategy and study selection

Working in collaboration with a specialist medical librarian, the study lead (HR) devised a comprehensive search strategy tailored to key databases using keywords, thesauri terms and Boolean Operators (Supplementary 1). Strategies included the MeSH terms ‘hypox*’, ‘cancer’, ‘neoplasm’, ‘lipid*’ and ‘fatty acid*’. MEDLINE, Embase and ISI Web of Science were searched from their inception to 31/08/2025. Supplementary grey literature was identified using OpenGrey. Reference lists from included papers were reviewed using a ‘snowball search’ methodology to identify relevant studies [27].

### Study selection

The inclusion criteria outlined that each paper must:

- Be original research.
- Investigate hypoxia and lipid metabolism within the context of cancer.
- Utilise in vitro, animal or human models.
- Induce physiological hypoxia by reducing environmental oxygen concentrations or confirm physiological tissue hypoxia using established measures of hypoxia (e.g. pimonidazole).
- Be written in English.
- Be published in peer-reviewed format (i.e. journal).

Studies were excluded if they were outside the scope of the review, not published in peer-reviewed literature (e.g. conference abstracts), or used hypoxia-mimetic agents (e.g. cobalt chloride) to artificially induce hypoxic pathways.

Screening was performed in two stages, ‘title and abstract screening’ and ‘full text review’, by three independent reviewers (HR, PL, and DC). Studies were included if they met the inclusion criteria. Any disagreement was resolved by discussion. Inter-rater agreement was assessed using Cohen’s Kappa [28]. Screening was performed using the Covidence software platform as recommended by the Cochrane Collaboration (Veritas Health Innovation, Melbourne) [29].

### Data extraction

For data extraction, an extensive data charting tool was utilised. Relevant data including author(s), year of publication, PubMed ID (PMID), cancer of interest, hypoxic conditions, pathway(s) of interest, downstream target/effect and experimental model were recorded. Hypoxic conditions were catalogued by hypoxic severity, length of exposure and type of hypoxia, i.e. acute (less than 24 h), chronic (equal or greater than 24 h) and cycling [30]. Where possible, quantitative analysis was performed to summarise existing research. To enable a thorough narrative summary, a qualitative thematic analysis was performed. Studies were grouped based on features of cancer lipid metabolism: (i) lipid accumulation, (ii) lipolysis, (iii) membrane metabolism, and (iv) lipid signalling. Where appropriate, subcategories were utilised. Two reviewers (HR and PL) extracted data independently. Figures were created in Biorender to synthesise the signalling pathways identified through thematic analysis and potential therapeutic interventions (Figs. 2, 3, 4, 5 and 6).

## Results

### Quantitative results

#### Included studies

**Fig. 1:**
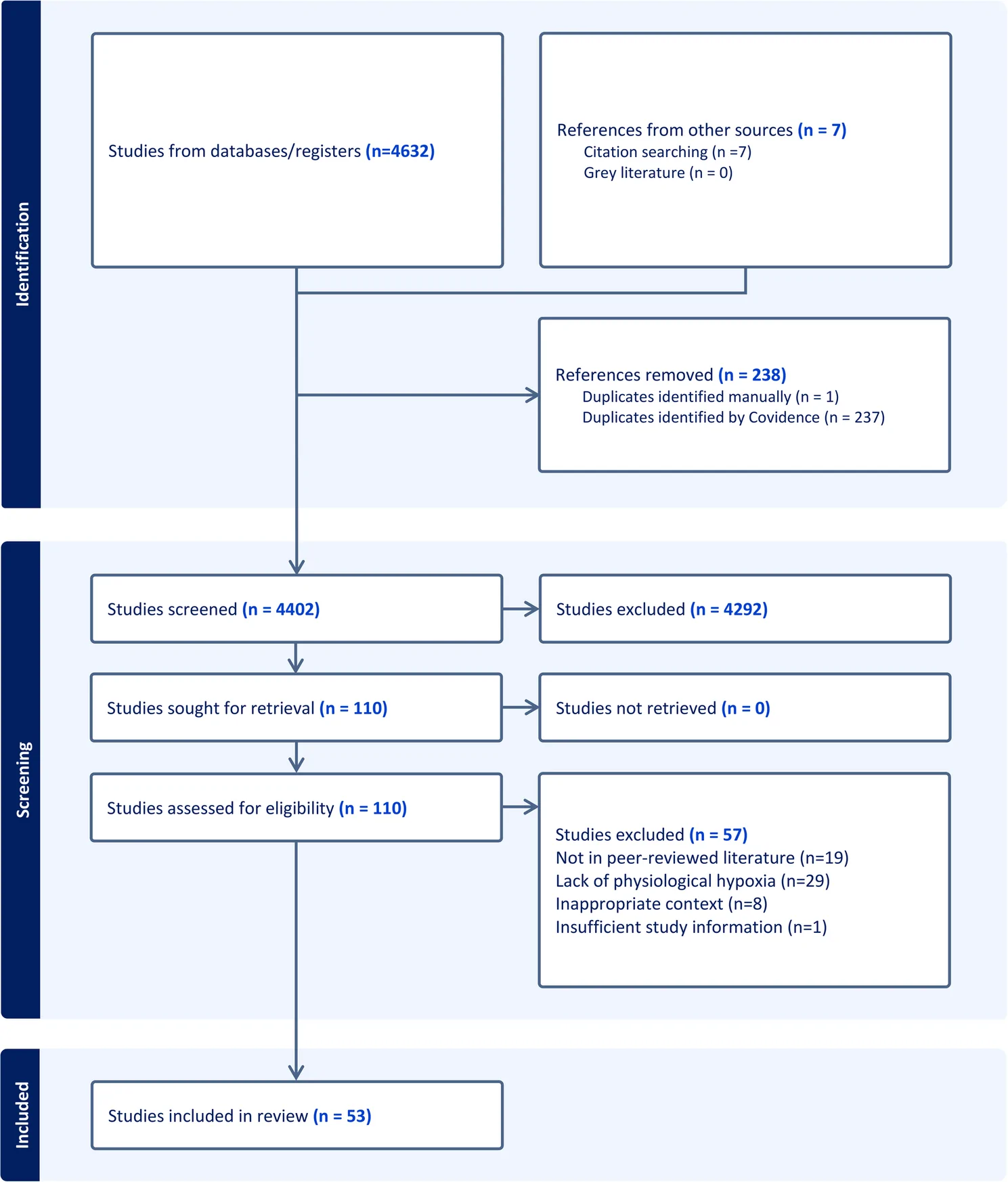
PRISMA Flowchart: Diagram summarising the number of records identified, screened, assessed for eligibility and included in the final review, with reasons for exclusion at the full-text stage. PRISMA, Preferred Reporting Items for Systematic Reviews and Meta-Analyses

After duplicate removal, the search identified 4632 studies for screening. Following abstract screening, 110 papers were brought forward to full text review. Of these, 46 were included for final analysis. After ‘snowball searching’ reference lists of included studies, 7 further articles were added. The most common reasons for exclusion at full text review were failure to publish in peer-reviewed literature (*n* = 19), lack of physiological hypoxia (*n* = 29) and non-cancer context (*n* = 7) (Fig. 1). No additional papers were identified from the grey literature search.

### Study characteristics

All 53 included studies were published in the last 20 years, with 2007 being the earliest publication (Table 1) [31–83]. A breadth of cancers was explored, with hepatocellular carcinoma (HCC) (*n* = 8, 15%), clear cell renal cell carcinoma (ccRCC) (*n* = 6, 11%) and colorectal cancer (*n* = 6, 11%) the most frequently individually studied diseases. Of all identified studies, 12 (23%) papers investigated multiple cancer types. When categorised by tumour lineage, sarcoma models were absent from the included literature (*n* = 0). Among single-cancer studies, most investigated carcinoma or carcinoma-derived tumour types (*n* = 36, 68%), with the remaining non-carcinoma studies comprising melanoma (*n* = 2, 4%) and glioblastoma (*n* = 3, 6%) models. Researchers frequently utilised immortalised commercial 2D cancer cell lines (*n* = 52, 98%). Other experimental models were spheroids (*n* = 7, 13%), mouse xenografts (*n* = 6, 11%) and primary culture (*n* = 2, 4%). For in vitro experiments, hypoxic chambers (HC) were used to control oxygenation (*n* = 53, 100%) and pimonidazole was used to confirm tissue hypoxia within in vivo specimens (*n* = 4, 8%).

**Table 1:** Study characteristics of included studies

Author, Year	PMID	Cancer	Hypoxic Exposure	Hypoxia Time (Hours)	Hypoxic O2	Control	Hypoxia Induced	Model	Pathway(s)	Target	Lipid Metabolism	Subtheme
Gao et al., 2016 [31]	27,357,947	HCC	Chronic	24	1%	21%	HC	2D	ACSS1/2	Acetate	Lipid Accumulation	De Novo Lipogenesis
Furuta et al., 2008 [32]	18,281,474	Breast	Chronic	48	1%	21%	HC, Pimonidazole	2D, Xenograft	Akt, SREBP1, HIF1	FASN	Lipid Accumulation	De Novo Lipogenesis
Jung et al., 2012 [33]	22,786,746	HCC	Acute	12	2%	21%	HC	2D	FASN	SREBP1	Lipid Accumulation	De Novo Lipogenesis
Scott et al., 2011 [34]	21,998,308	Melanoma	Chronic	24	1%	21%	HC	2D	HIF1	Glutamine	Lipid Accumulation	De Novo Lipogenesis
Husain et al., 2022 [35]	35,227,773	HCC	Chronic	24	1%	20%	HC	2D	HIF1, EphA3 EphA2	SREBP1, ACLY	Lipid Accumulation	De Novo Lipogenesis
Liu et al., 2021 [36]	34,098,990	ccRCC	Chronic	24	1%	21%	HC	2D	HIF1	GDP1	Lipid Accumulation	De Novo Lipogenesis
Wang et al., 2017 [37]	28,435,451	ccRCC	Chronic	48	1%	21%	HC	2D	HIF1	AHNAK2	Lipid Accumulation	De Novo Lipogenesis
Tsui et al., 2013 [38]	23,709,747	Prostate	Chronic	24	1%	21%	HC	2D	HIF1	mACON	Lipid Accumulation	De Novo Lipogenesis
Sun et al., 2014 [39]	24,506,869	Multiple	Acute	16	0.5%	21%	HC	2D	HIF1	SIAH2	Lipid Accumulation	De Novo Lipogenesis
Eguchi et al., 2019 [40]	31,582,221	Breast	Chronic	48	1%	21%	HC	2D	HIF1	PDH Complex	Lipid Accumulation	De Novo Lipogenesis
Choi et al., 2022 [41]	36,227,691	Gastric	Chronic	24	1%	21%	HC	2D	HIF1	Viperin	Lipid Accumulation	De Novo Lipogenesis
Filipp et al., 2012 [42]	22,360,810	Melanoma	Chronic	24	1%	21%	HC	2D	IDH1, IDH2	Glutamine	Lipid Accumulation	De Novo Lipogenesis
Huang et al., 2025 [43]	39,827,204	CRC	Chronic	24–72	1%	21%	HC	2D	SFRP2, ITLN1	SREBP1	Lipid Accumulation	De Novo Lipogenesis
Glowacka et al., 2024 [44]	39,572,570	Multiple	Chronic	24	1%	21%	HC, Pimonidazole	2D, Spheroid, Xenograft	SCD-1	MUFA	Lipid Accumulation	Exogenous uptake
Offer et al., 2019 [45]	31,174,567	GBM	Chronic	48	1%	21%	HC	2D	CREB	VEGF, HGF	Lipid Accumulation	Exogenous Uptake
Echeverri et al., 2021 [46]	34,749,809	Pancreatic	Acute	16	1%	21%	HC	2D	HIF1	PDH1	Lipid Accumulation	Exogenous Uptake
Valli et al., 2015 [47]	25,605,240	CRC	Chronic	24	1%	21%	HC	2D	HIF1	SREBP1, SCD-1, FASN	Lipid Accumulation	Exogenous Uptake
Shen et al., 2012 [48]	21,970,364	Multiple	Chronic	24	1%	21%	HC	2D	HIF1	VLDLR	Lipid Accumulation	Exogenous Uptake
Young et al., 2013 [49]	23,699,409	Multiple	Chronic	48	0.5%	21%	HC	2D	mTOR	UPR, IRE-1	Lipid Accumulation	Exogenous Uptake
Kamphorst et al., 2013 [50]	23,671,091	Multiple	Chronic	72	1%	21%	HC	2D	Ras, Akt	SCD-1	Lipid Accumulation	Exogenous Uptake
Seo et al., 2020 [51]	33,128,030	HCC	Chronic	24	1%	21%	HC	2D, Spheroid	FABP5	HIF1	Lipid Accumulation	Lipid Droplet
Seo et al., 2021 [52]	34,775,260	CRC	Acute	8	1%	21%	HC	2D, Spheroid	FABP5	HIF1	Lipid Accumulation	Lipid Droplet
Triantafyllou et al., 2018 [53]	29,908,837	Multiple	Acute & Chronic	8 and 24	1%	21%	HC	2D	HIF1	AGPAT2	Lipid Accumulation	Lipid Droplet
Shen et al., 2015 [54]	26,498,183	Breast	Chronic	24	1%	21%	HC	2D	HIF1	ADRP	Lipid Accumulation	Lipid Droplet
Kourti et al., 2015 [55]	25,744,540	Multiple	Chronic	24	1%	21%	HC	2D	HIF1	CK1d	Lipid Accumulation	Lipid Droplet
Bensaad et al., 2014 [56]	25,263,561	Multiple	Chronic	24,48,72	0.1%	21%	HC	2D, Spheroid	HIF1	FABP3, FABP7, ADRP	Lipid Accumulation	Lipid Droplet
Mylonis et al., 2012 [57]	22,467,849	Cervical	Chronic	24 and 48	1%	21%	HC	2D	HIF1	Lipin-1	Lipid Accumulation	Lipid Droplet
Laurenti et al., 2011 [58]	21,866,563	GBM	Chronic	96	1%	21%	HC	Primary Culture	HIF1	PPARa	Lipid Accumulation	Lipid Droplet
Gimm et al., 2010 [59]	20,624,928	Multiple	Acute	16	1%	21%	HC	2D	HIF1	HILPDA	Lipid Accumulation	Lipid Droplet
VandeKopple et al., 2019 [60]	31,308,147	CRC	Chronic	72	1%	21%	HC	2D	HIF1	HILPDA	Lipid Accumulation	Lipid Droplet
Chen et al., 2019 [61]	31,796,646	HCC	Chronic	24	1%	21%	HC	2D	HIF2	mTOR	Lipid Accumulation	Lipid Droplet
Qui et al., 2015 [62]	25,829,424	ccRCC	Chronic	24	0.5%	21%	HC, Pimonidazole	2D, Spheroid, Xenograft	HIF2, UPR	ADPR	Lipid Accumulation	Lipid Droplet
Menard et al., 2016 [63]	27,199,348	GBM	Chronic	72	1%	21%	HC	2D, Spheroid	HSPG	ERK, MAPK	Lipid Accumulation	Lipid Droplet
Ackerman et all, 2018 [64]	30,184,495	ccRCC	Chronic	72	0.5%	21%	HC	2D	SCD-1	DGAT	Lipid Accumulation	Lipid Droplet
Genard et al., 2024 [65]	39,257,626	CRC	Chronic	72	2%	21%	HC	Primary culture	RAB 5 C	LDs, sEVs	Lipid Accumulation	Lipid Droplet
Gonzalez et al., 2025 [66]	39,886,224	PDAC	Chronic	24–72	0.1%	21%	HC	2D	HIF	UPR	Lipid Accumulation	Lipid Droplet
Sainero-Alcolado et al., 2024 [67]	38,335,255	ccRCC	Chronic	72	1%	21%	HC	2D, Xenograft	MYC, HIF2	HILDPA	Lipid Accumulation	Lipid Droplet
Zhang et al., 2017 [68]	29,256,392	Multiple	Chronic	24	0.5%	20%	HC	2D	HIF1a	HILDPA, ATGL	Lipid Accumulation	Lipid Droplet
MacVicar et al., 2019 [69]	31,695,197	Pancreatic	Chronic	24	0.5%	21%	HC	2D, Spheroid	HIF, mTOR	YME1, Lipin1	Lipid Signalling	Tumourogenicity
Hart et al., 2019 [70]	30,655,321	Ovarian	Chronic	48	1%	21%	HC	2D	HIF1, HIF2	SPHK1	Lipid Signalling	Tumourogenicity
Ni et al., 2020 [71]	33,059,020	Lung	Chronic	48	1%	21%	HC	2D	mIR-15a-5p	ACSS2	Lipid Signalling	Tumourogenicity
Du et al., 2017 [72]	29,176,561	ccRCC	Chronic	24	1%	21%	HC	2D	HIF1	CPT1A	Lipolysis	Fatty Acid Oxidation
Huang et al., 2014 [73]	25,242,319	HCC	Chronic	48	1%	20%	HC	2D	HIF1	MCAD, LCAD	Lipolysis	Fatty Acid Oxidation
Saliakoura et al., 2020 [74]	33,077,911	Lung	Chronic	48	1%	21%	HC, Pimonidazole	2D Xenograft	HIF1	PLCy1	Lipolysis	Fatty Acid Oxidation
Liu et al., 2014 [75]	24,503,013	HCC	Acute	18	1%	21%	HC	2D	HIF1, HIF2	PGC-1a, CPT-1	Lipolysis	Fatty Acid Oxidation
Zhou et al., 2024 [76]	38,996,932	PDAC	Chronic	96	1%	20%	HC	2D	YY1	CPT1C	Lipolysis	Fatty Acid Oxidation
Zhang et al., 2025 [77]	39,947,475	Multiple	Chronic	24, 72	1%	21%	HC	2D	HIF1	CPT1C, MED15	Lipolysis	Fatty Acid Oxidation
Jung et al., 2025 [78]	39,975,955	Breast	Chronic	48	1%	21%	HC	2D	SREBP1	ACADVL, ACADM, HADHA	Lipolysis	Fatty Acid Oxidation
Liang et al., 2024 [79]	38,450,796	Thyroid	Acute & Chronic	12–48	1%	21%	HC	2D, Xenograft	HIF1	CPT1A	Lipolysis	Fatty Acid Oxidation
Kiraly et al., 2013 [80]	24,317,474	Multiple	Chronic	96	1%	21%	HC	2D	FA2H	-	Membrane Metabolism	Cell
Bansal et al., 2012 [81]	22,025,560	Prostate	Chronic	24	1%	21%	HC	2D	HIF1	ChKa	Membrane Metabolism	Cell
El Hindi, et al. 2024 [82]	37,802,156	CRC	Chronic	24–72	1%	21%	HC	2D	CerS4, CerS5	-	Membrane metabolism	Cell
Bao et al., 2021 [83]	33,503,428	HCC	Chronic	168	1%	20%	HC	2D	PTPMT1	Cardiolipin	Membrane Metabolism	Mitochondrial

Most papers studied the effect of chronic hypoxia (*n* = 45, 85%). Just 6 (11%) publications reviewed acute hypoxia and 2 (4%) papers studied both acute and chronic hypoxic environments. Cycling hypoxic conditions were unexplored. In the acute hypoxia setting, the most frequently utilised timeframe was 16 h (*n* = 3, 5%). For the chronic group, 24 h was the most common timepoint (*n* = 19, 36%), followed by 48 (*n* = 10, 19%) and 72 h (*n* = 6, 11%). The longest exposure period was 7 days. In terms of hypoxic severity, 81% (*n* = 43) of articles studied moderate hypoxia (1–2% O_2_) [84]. A small selection (*n* = 8, 15%) investigated severe or radiobiological hypoxic conditions (0.1–0.5% O_2_). Control conditions were unanimously either 21% or 20% oxygenation, therefore the impact of physiological tissue normoxia, termed oxic conditions (from 5 to 10% O_2_), was not demonstrated in any study [6].

### Pathways, targets and effectors

Unsurprisingly, HIF was the dominant pathway of interest and the focus of 35 (66%) manuscripts. HIF-1 was more prominent and appeared 31 (58%) times as the parent pathway and twice (4%) as a downstream target. HIF-2 was only reported 5 (9%) times as the primary pathway. Aside from HIF, a vast array of alternative pathways, downstream targets and effectors were suggested to be important in hypoxic lipid metabolism. Of note, both the Unfolded Protein Response (UPR) pathway and Mammalian Target of Rapamycin (mTOR) pathway were a focus of 3 (6%) papers. The most frequently discussed targets and effectors included Sterol Regulatory Element-Binding Protein-1 (SREBP-1) (*n* = 6, 11%), Hypoxia-Inducible Lipid Droplet-associated (HILPDA) (*n* = 5, 9%), Carnitine Palmitoyltransferase-1 (CPT1) (*n* = 5, 9%), Stearoyl-CoA Desaturase-1 (SCD-1) (*n* = 4, 8%), Fatty Acid Binding Proteins (FABP) (*n* = 3, 6%) and Fatty Acid Synthase (FASN) (*n* = 3, 6%).

### Thematic analysis

Studies were grouped by their functional contribution to alternate components of cancer lipid metabolism. Lipid accumulation was the largest category (*n* = 38, 72%) and was subdivided into lipid droplet (LD) formation (*n* = 18, 34%), de novo lipogenesis (*n* = 13, 25%) and exogenous lipid uptake (*n* = 7, 13%). A total of 8 (15%) studies were grouped under lipolysis and similarly 3 (6%) papers discussed lipid signalling. Finally, 4 (8%) articles outlined hypoxic membrane metabolism and were subcategorised into cell membrane (*n* = 3, 6%) and mitochondrial membrane (*n* = 1, 2%).

### Qualitative narrative review

#### Lipid accumulation

**Fig. 2:**
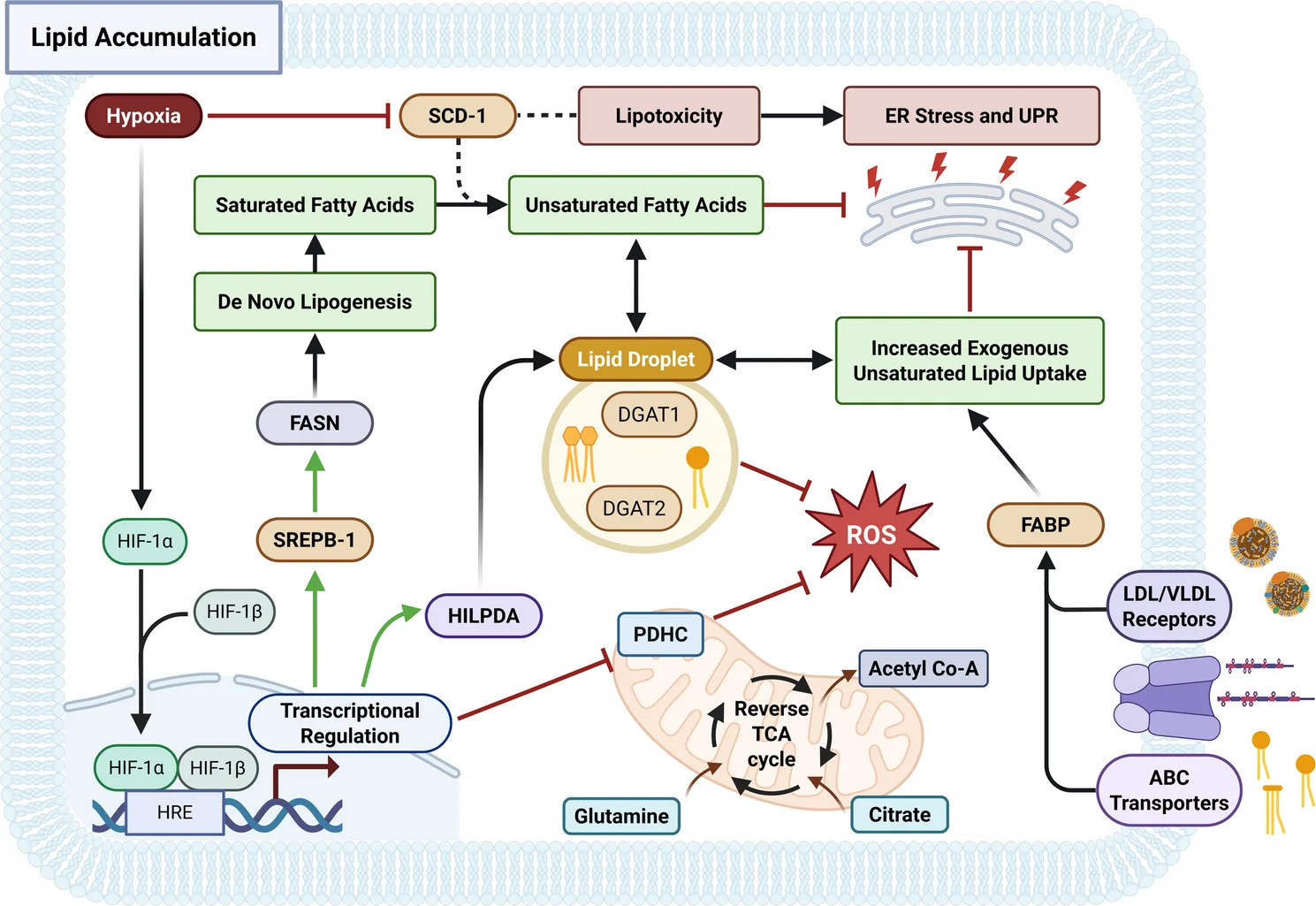
Lipid Accumulation: Tumour hypoxia stabilises HIF-1α/HIF-1β and activates transcriptional programmes that promote lipid accumulation. HIF-driven regulation of SREBP-1 and FASN generates saturated fatty acids through de novo lipogenesis. With SCD-1 downregulated in hypoxia, there is an increased reliance on unsaturated fatty acids from lipid droplets and exogenous uptake. Hypoxia rewires mitochondrial metabolism, including altered PDHC-complex activity and glutamine-dependent citrate production, to support acetyl-CoA generation for lipid synthesis. Together, these adaptations reduce lipotoxicity, ROS, and ER stress, supporting cancer cell survival under hypoxia

The literature identified in this scoping review demonstrates that hypoxia drives a lipogenic cancer phenotype through multiple mechanisms, conferring crucial survival advantages [47]. Under hypoxic conditions, cancer cells undergo profound metabolic reprogramming involving de novo lipogenesis, LD formation and exogenous lipid uptake (Fig. 2).

In healthy states, *de novo* lipogenesis is largely restricted to hepatocytes and adipocytes, where fatty acids are synthesised using carbon derived from carbohydrates and amino acids. As outlined, upon malignant transformation, *de novo* lipogenesis is activated to produce essential nutrients for growth and survival [85]. Whilst hypoxic cancer cells similarly engage *de novo* lipogenesis, the underlying mechanisms differ, and their reliance on this process is debated.

Hypoxia alters de novo lipogenesis by driving cancer cells to seek alternative carbon sources. This lowers their dependence on mitochondrial oxidative metabolism, reducing oxygen consumption and aiding cell survival. Repeatedly, HIF-1 has been found to induce a metabolic switch from glucose to glutamine as a primary carbon source for fatty acid biosynthesis [34, 39, 42]. Through the ‘reverse’ tricarboxylic acid (TCA) cycle, citrate is preferentially generated from glutamine via reductive carboxylation, a process catalysed by isocitrate dehydrogenase (IDH)-1 and IDH-2. The resulting Acetyl-CoA can then be used for fatty acid synthesis. Likewise, Gao et al. have outlined that hypoxic conditions increase the uptake of acetate, which is employed as a carbon source for acetyl-CoA and lipid production [31]. Interestingly, acetate also potentiates this process by increasing histone acetylation, thereby epigenetically activating lipogenic genes including FASN [31]. These alternative carbon sources collectively attenuate mitochondrial oxidative metabolism, limit cellular oxygen demand and enable de novo lipogenesis.

This is crucial for cell survival in an oxygen-deprived environment. It has been well-described that mitochondria are the primary site of ROS generation [86]. Under hypoxic conditions, the electron transport chain is impaired, exacerbating electron leak from Complex III and mitochondrial ROS production [87–89]. ROS accumulation can become intolerable and pro-apoptotic in cancer cells, through numerous mechanisms including irreversible DNA damage and triggering apoptotic pathways such as c-Jun N-terminal kinase (JNK) signalling [90]. Therefore, it could be inferred that by utilising alternative carbon sources for de novo lipogenesis and limiting mitochondrial oxidative metabolism, toxic ROS accumulation is potentially prevented.

The importance of de novo lipogenesis in hypoxic tumours is debated. For example, in the context of HCC, HIF-dependent lipogenic transcription factors, proteins and enzymes, including SREBP-1, ATP citrate lyase (ACLY) and FASN, are essential for cancer cell survival [35, 78]. In breast cancer, Furuta et al. demonstrated *de novo* lipogenesis stimulated by Akt/HIF-1-dependent activation of the SREBP-1/FASN axis was integral for hypoxic cancer cell proliferation [32]. In contrast, Eguchi et al. found HIF-1α inhibits pyruvate dehydrogenase (PDH) complex and therefore directly limits potential lipogenesis [40]. Similarly, in ccRCC, HIF-1α positively regulates glycerol-3-phosphate dehydrogenase 1 (GDP1), which then downregulates Prolyl Hydroxylase Domain 3 (PHD3), thus amplifying HIF-1α and creating a positive feedback loop. This loop decreases the expression of GDP2, inhibits mitochondrial function and suppresses lipid metabolism [36]. These contrasting findings indicate the significance of hypoxic tumour *de novo* lipogenesis is context-dependent and tissue-specific.

This context dependency is consistent with broader cancer lipid metabolism, where reliance on *de novo* lipogenesis varies by tissue lineage, oncogenic driver, nutrient availability and the capacity to acquire exogenous lipids [91]. SREBP-regulated lipogenic pathways involving ACLY, ACC, FASN and SCD are frequently activated in cancer, but their functional importance is not uniform across tumour types or microenvironmental states. Therefore, the effect of hypoxia on de novo lipogenesis should be interpreted as context-specific rather than universally pro- or anti-lipogenic [92–95].

Hypoxia-driven lipid metabolism intersects closely with glycolysis, glutaminolysis and mitochondrial metabolism [9]. HIF-mediated enhancement of glycolysis reduces reliance on mitochondrial glucose oxidation, while glutamine can be redirected through reductive carboxylation to sustain citrate and acetyl-CoA production for fatty acid synthesis under oxygen-limited conditions [34, 39, 42]. Similarly, acetate uptake provides an additional carbon source for acetyl-CoA and lipogenic gene regulation under hypoxia [31]. These interactions illustrate how lipid metabolism is integrated with broader metabolic rewiring, allowing hypoxic cancer cells to maintain lipid synthesis and membrane homeostasis while limiting oxygen consumption and mitochondrial ROS generation [86–90].

Lipid droplets (LDs) are cellular organelles consisting of a neutral lipid core surrounded by a single phospholipid membrane [96]. As a lipid reservoir, they are integral for lipid homeostasis, energy metabolism and cellular signalling. Many identified articles highlighted the importance of LDs for cancer cell survival under hypoxic conditions [51–68]. In hypoxia, both HIF-1 and HIF-2 contribute to LD formation, although the downstream mechanisms appear context dependent [51–62, 66–68].

HIF-1α has been linked to lipid loading through several complementary mechanisms, including Fatty Acid Binding Protein (FABP)-mediated fatty acid handling, lipin-1-dependent neutral lipid synthesis, 1-acylglycerol-3-phosphate O-acyltransferase 2 (AGPAT2)-dependent glycerophospholipid and triacylglycerol biosynthesis, and VLDLR-mediated lipoprotein uptake [48, 51–57]. Downstream of HIF activation, Hypoxia-Inducible Lipid Droplet Associated (HILPDA), also known as Hypoxia-Inducible Protein 2, further regulates LD abundance, lipid storage and adaptation to microenvironmental stress [59, 60, 67, 68].

HIF-2α appears particularly relevant in clear-cell renal cell carcinoma, where HIF-2α-dependent induction of PLIN2 promotes lipid storage, endoplasmic reticulum homeostasis and tumour cell survival [62]. Furthermore, Sterol O-acyltransferase 1 (SOAT1) can esterify free cholesterol into cholesteryl esters for LD storage. As an intracellular cholesterol acyltransferase, SOAT1 esterifies free cholesterol with long-chain fatty acyl-CoAs to generate cholesteryl esters, which are stored within the neutral lipid core of LDs [97]. This process can buffer excess free cholesterol, reduce cholesterol-associated membrane or ER stress, and support neutral lipid accumulation [98]. Although direct HIF-dependent regulation of SOAT1 has not been consistently established across tumour types, hypoxia-driven changes in lipid uptake, SREBP-associated lipogenic programmes and LD biogenesis may converge on SOAT1-dependent cholesterol esterification in selected tumour contexts [47, 56, 59, 62, 99, 100]. Further work is required to define whether SOAT1 is directly regulated by hypoxia or functions as a downstream component of broader hypoxia-associated adaptations in cholesterol handling and lipid storage.

The lipogenic cancer phenotype can be self-destructive as the primary lipid desaturase enzyme, Stearoyl-CoA desaturase-1 (SCD-1), is oxygen-dependent [44, 47, 50, 64]. With SCD-1’s activity inhibited by hypoxia, cancer cells are unable to convert saturated fatty acid (SFAs) derived from *de novo* lipogenesis, such as palmitate (16:0) and stearate (18:0), into essential monounsaturated fatty acids (MUFAs) like palmitoleic acid (16:1) or oleic acid (18:1) [50]. This disrupts the lipid desaturation index, driving pro-apoptotic lipotoxicity as well as compromising membrane fluidity and cell integrity [50, 59, 60]. LDs mitigate lipotoxicity through several protective mechanisms. First, they can sequester SFA and serve as dynamic reservoirs capable of releasing mono- and polyunsaturated fatty acids from triglyceride stores to restore the cellular lipid saturation index [57, 60, 62, 66]. Furthermore, LDs shield unsaturated lipids from elevated ROS in the hypoxic cell cytoplasm to limit lipid peroxidation [74]. By storing fatty acids, LDs reduce ROS generation associated with lipolysis and provide metabolic plasticity [68, 77]. LDs are integral to hypoxic cancer lipid metabolism, as when LD formation is impaired, hypoxia exacerbates endoplasmic reticulum (ER) stress, initiates the Unfolded Protein Response (UPR) and induces apoptosis [49, 62, 64, 66].

Hypoxic cancer cells are reliant on exogenous lipid uptake [44–50]. HIF activation facilitates lipid loading by upregulating membrane transporters from the ATP-binding cassette (ABC) superfamily, as well as low- and very-low-density lipoprotein receptors (LDLR/VLDLR) [47, 48]. Metabolic flux experiments highlight that hypoxic cancer cells preferentially take up unsaturated fatty acids, which help restore the desaturation index, and phospholipids with one fatty acid tail (lysophospholipids) that can be readily utilised to restore membrane fluidity [50]. When *de novo* synthesis of unsaturated fatty acids is inhibited, as in the hypoxic tumour environment, exogenous desaturated lipid uptake is critical to alleviate ER stress and reverse Inositol-Requiring Enzyme-1 (IRE-1) dependent cell death [49]. Interestingly, Głowacka et al. describe a metabolic interplay in which acid-adapted tumour cells adjacent to hypoxic niches exhibit elevated SCD-1 activity and enhanced production of MUFAs, thereby supporting the survival of neighbouring hypoxic cells [44]. Collectively, these studies underscore the dependence of hypoxic cancer cells on abundant exogenous, particularly desaturated, lipids and highlight the metabolic cooperation between distinct tumour microenvironmental niches.

### Lipolysis

**Fig. 3:**
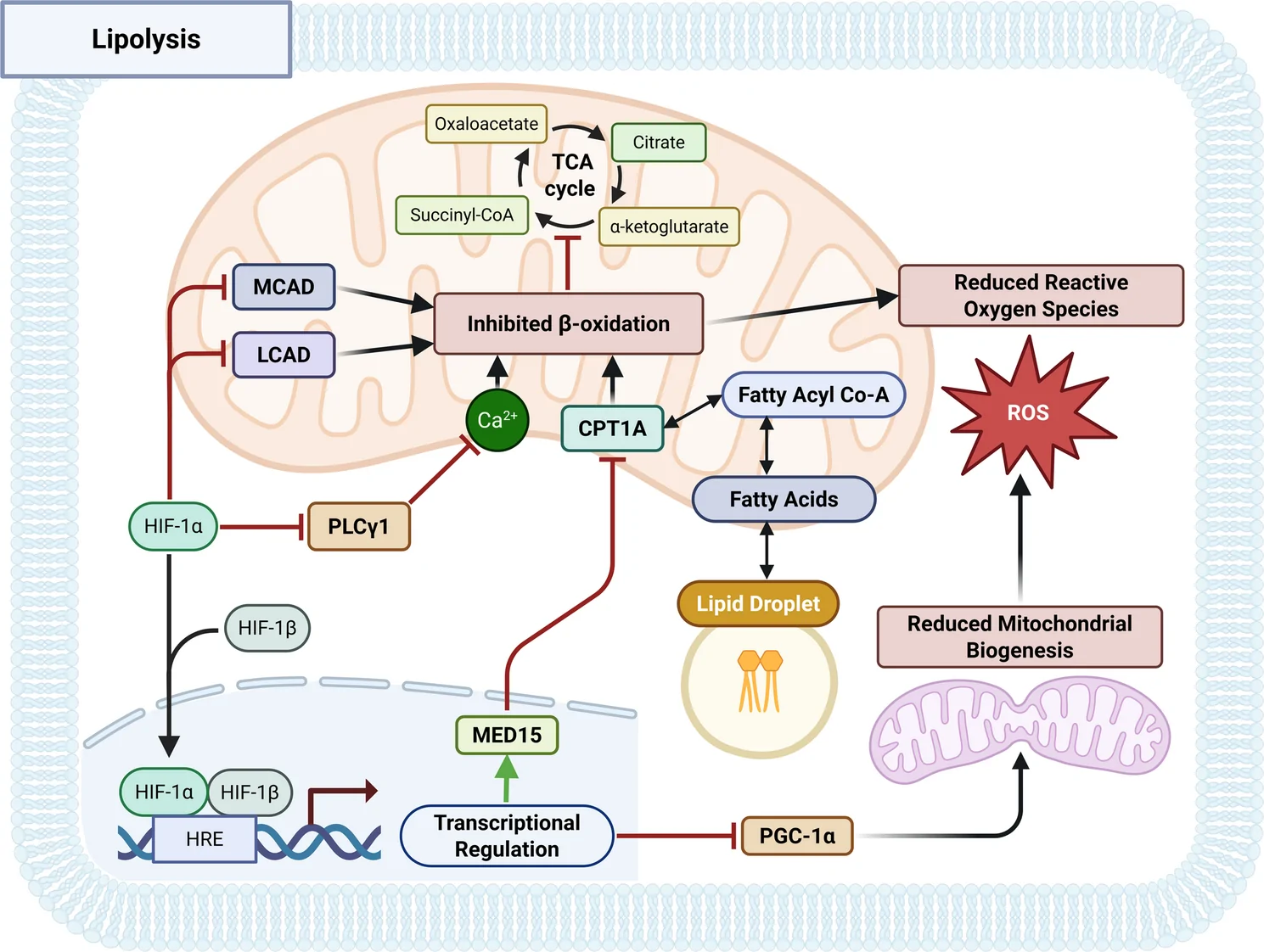
Lipolysis: Tumour hypoxia stabilises HIF-1α/HIF-1β and activates transcriptional programmes that inhibit mitochondrial fatty acid oxidation. HIF-dependent regulation of PLCγ1, MED15, CPT1A, MCAD, and LCAD limits fatty acid entry into β-oxidation and suppresses TCA cycle activity. Reduced mitochondrial biogenesis through PGC-1α further decreases oxidative metabolism. As fatty acids are diverted away from mitochondrial oxidation, they accumulate in lipid droplets, while reduced β-oxidation and mitochondrial activity help limit ROS production

In the hypoxic tumour microenvironment, lipolysis is frequently constrained to minimise oxygen utilisation and limit redox stress (Fig. 3). For example, in renal, liver, lung, pancreatic and colorectal cancers, fatty acid oxidation (FAO) is inhibited via numerous HIF-dependent mechanisms [68, 72–76]. Specifically, HIF signalling downregulates carnitine palmitoyltransferase 1 A (CPT1A), the rate-limiting enzyme responsible for fatty acid import into the mitochondria, thereby reducing FAO [72, 75]. Additionally, it directly inhibits oxidative enzymes including medium- and long-chain acyl-CoA dehydrogenases (MCAD and LCAD), further constraining FAO [73]. HIF-1α and HIF-2α also suppress the transcription factor peroxisome proliferator-activated receptor gamma coactivator 1α (PGC-1α), curbing mitochondrial biogenesis and FAO capacity [75]. Collectively, suppression of FAO under hypoxia reduces potential lipid peroxidation from oxidative stress and promotes protective lipid accumulation.

Select studies had contradictory findings. Liang et al. reported that papillary thyroid carcinoma cells exposed to hypoxic stress upregulate CPT1A [79], resulting in increased FAO, reduced lipid accumulation, enhanced expression of the antioxidant enzyme superoxide dismutase (SOD) and conserved mitochondrial integrity. These adaptations lower cellular ROS and encourage proliferation. In triple negative breast cancer (TNBC) cells, chronic hypoxia induces SREBP-1, lipophagy and downstream FAO through oxidative enzymes, including Very long-chain acyl-CoA Dehydrogenase (ACADVL), Medium-Chain Acyl-CoA Dehydrogenase (ACADM) and Hydroxyacyl-CoA Dehydrogenase trifunctional multienzyme complex subunit alpha (HADHA) [78]. These conflicting findings emphasise the dynamic and heterogenous nature of hypoxic metabolic reprogramming, which is highly dependent on tumour type.

### Membrane metabolism

**Fig. 4:**
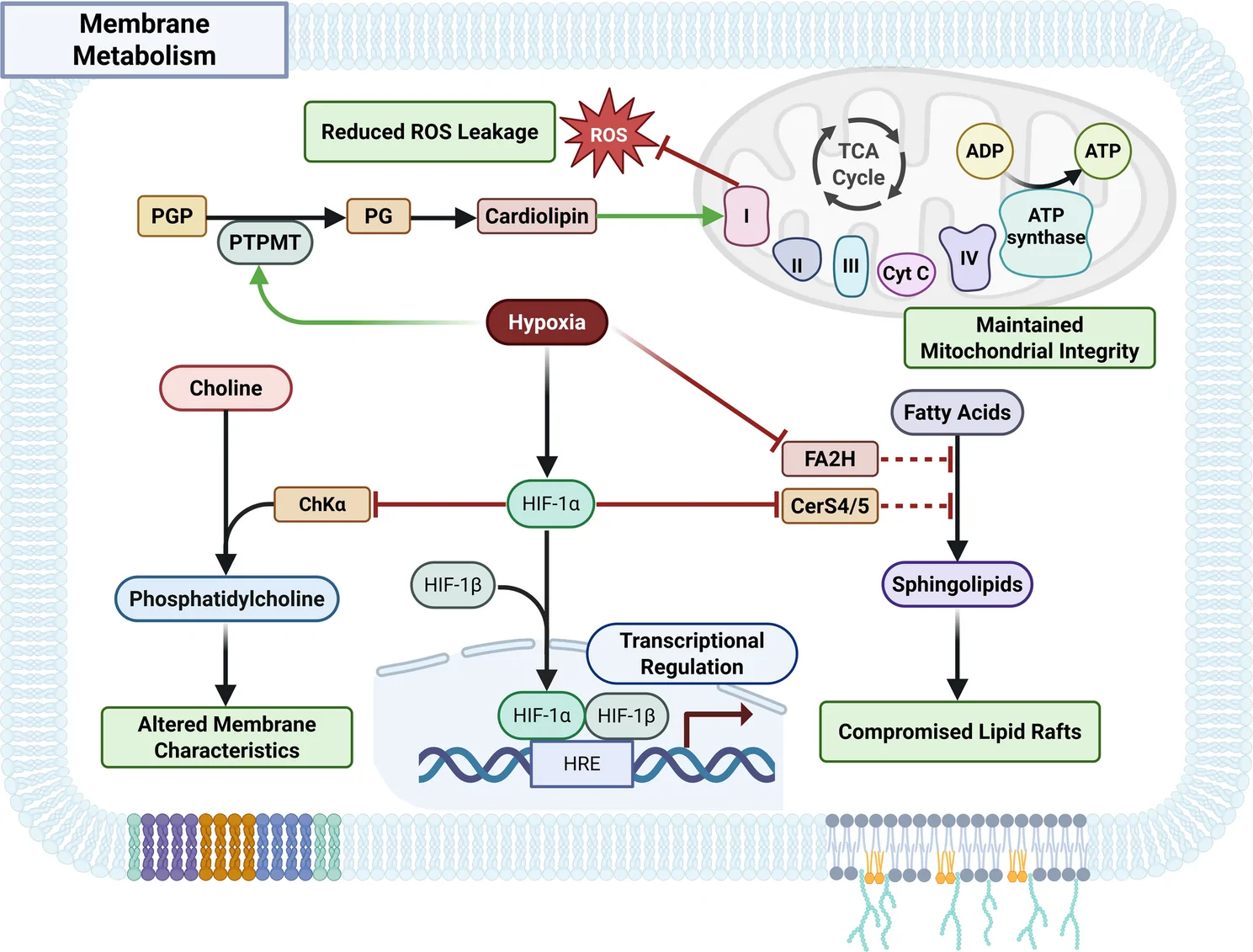
Membrane Metabolism: Tumour hypoxia stabilises HIF-1α/HIF-1β and alters membrane lipid metabolism through transcriptional and enzymatic regulation. Hypoxia promotes PTPMT1-dependent cardiolipin remodelling, helping preserve mitochondrial integrity and reduce ROS leakage. In parallel, HIF-1α suppresses choline kinase activity, altering phosphatidylcholine synthesis and the cell membrane. Hypoxia inhibits FA2H and CerS4/5, disrupting sphingolipid metabolism and compromising lipid raft organisation

Hypoxia disrupts the phospholipid membrane of cancer cells and their mitochondria (Fig. 4) [80–83]. These changes are mediated through both HIF-independent and HIF-dependent processes. For instance, fatty acid 2-hydroxylase (FA2H), an oxygen-dependent enzyme important for plasma membrane integrity, exhibits reduced activity within the hypoxic tumour microenvironment, leading to altered membrane fluidity and clustering of lipid rafts [80]. Similar hypoxia-driven changes in membrane properties and lipid raft dynamics have been reported in colorectal cancer [82]. These effects are closely linked to cholesterol metabolism, as cholesterol is a major determinant of membrane order, rigidity, permeability and lipid raft organisation. Cholesterol-rich raft domains provide platforms for receptor localisation and oncogenic signalling, but their increased cholesterol content can also reduce local membrane fluidity [101]. Hypoxia-associated changes in lipid uptake, sterol handling and cholesterol esterification may therefore alter membrane structural integrity by reshaping lipid raft abundance, membrane rigidity and transporter or receptor distribution. In this context, hypoxia-driven disruption of cholesterol-rich membrane domains may contribute to altered drug uptake, survival signalling and treatment resistance [80, 82].

Through radiolabelled choline tracer experiments, HIF has been shown to attenuate choline kinase-α (ChKα), a key component of membrane phospholipid synthesis, reducing cell membrane biosynthesis. Resulting disordered ‘leaky’ membranes may confer a survival advantage under hypoxia by facilitating lipid uptake [45]. Hypoxia also imposes substantial stress on mitochondrial membranes, necessitating protective adaptations. Bao et al. demonstrated protein-tyrosine phosphatase mitochondrial 1 (PTPMT1), a critical enzyme for cardiolipin synthesis, is a key determinant of hypoxic cell survival. Cardiolipin is an essential constituent of the inner mitochondrial membrane responsible for assembly and stabilisation of electron transport chain (ETC) complexes. Loss of PTPMT1 impairs cardiolipin maturation, exacerbates electron leakage from the ETC and increases ROS accumulation, ultimately compromising cell survival. Together these findings highlight how hypoxia-driven lipid reprogramming reshapes cellular and mitochondrial membranes whilst preserving mitochondrial integrity.

### Lipid signalling

**Fig. 5:**
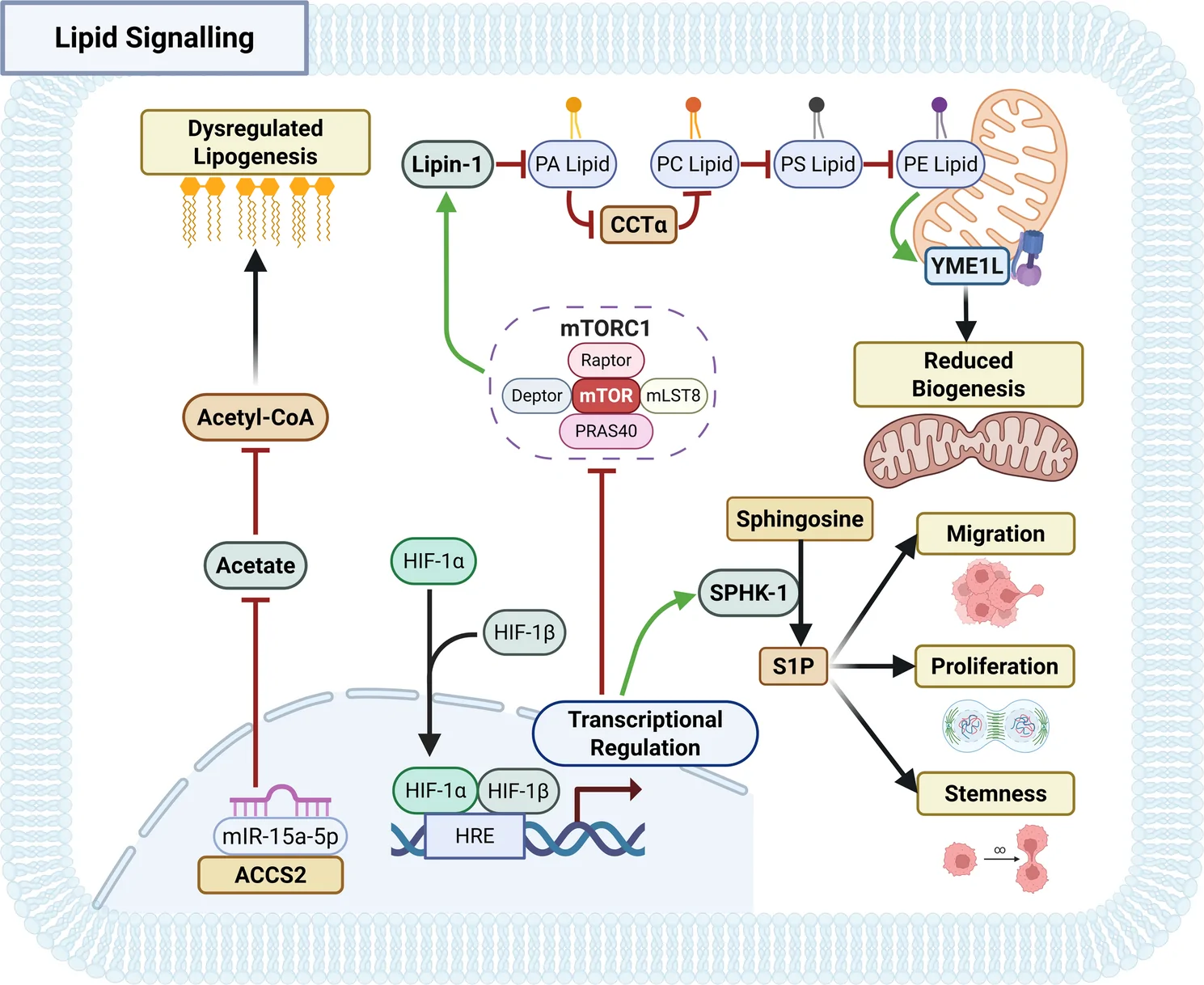
Lipid Signalling: Tumour hypoxia promotes HIF binding and rewires lipid signalling pathways that support malignant phenotypes. HIF-dependent transcriptional regulation modulates mTORC1, lipin-1 and YME1L, reducing mitochondrial biogenesis. Hypoxia also promotes SPHK-1-mediated conversion of sphingosine to S1P, enhancing migration, proliferation, and stemness

Beyond serving as nutrients and structural components, lipids also function as signalling molecules and play important roles in hypoxia-driven tumourigenesis (Fig. 5) [69–71]. For example, in pancreatic cancer, MacVicar et al. described that HIF-1-mediated inhibition of mTORC1 initiates a lipid signalling cascade that promotes YME1L-mediated mitochondrial proteolysis and suppresses mitochondrial biogenesis [69]. This pathway is proposed to limit mitochondria-derived pro-apoptotic stress while redirecting cellular resources towards growth. Alongside this, HIF stimulates the activity of sphingosine kinase-1 (SPHK-1), a key enzyme in the production of the bioactive lipid sphingosine-1-phosphate (S1P) [70]. S1P has wide-ranging effects including the promotion of proliferation, invasion and stemness. From a regulatory perspective, Ni et al. identified a tumour-suppressive role for microRNA miR-15a-5p, which translocates to the nucleus under hypoxic conditions to impair lipogenesis by suppressing acetyl-CoA synthetase 2 (ACSS2), reducing acetate uptake and inhibiting the transcription of FASN and Acetyl-CoA Carboxylase (ACC) via histone H4 acetylation [71]. The resulting compromised lipid synthesis restricts lung cancer growth and metastatic potential, illustrating a novel future therapeutic target.

### Baseline lipid metabolic programmes in cancer

Under non-hypoxic growth conditions, cancer cells frequently display enhanced lipid biosynthesis, uptake and storage to support proliferation, membrane biogenesis, redox balance and oncogenic signalling [85]. De novo lipogenesis is commonly coordinated through SREBP-regulated transcriptional programmes involving ATP citrate lyase (ACLY), acetyl-CoA carboxylase (ACC) and fatty acid synthase (FASN), which convert citrate-derived carbon into fatty acids for phospholipid, triglyceride and signalling lipid production. In parallel, lipid storage and membrane homeostasis are influenced by fatty acid desaturation, cholesterol handling and lipid droplet-associated pathways, including enzymes such as SCD-1 and SOAT1 in relevant tumour contexts [17]. Against this baseline, the studies included in this review indicate that hypoxia can remodel lipid acquisition, synthesis, storage and oxidation in a context-dependent manner, influenced by tumour type, HIF isoform activity and availability of exogenous lipid substrates [31, 47, 56, 72].

## Discussion

To the authors’ knowledge, this is the first scoping review synthesising the current understanding of the hypoxic tumour microenvironment and its multifaceted reprogramming of lipid metabolism in cancer cells. The scoping review methodology allows for a breadth of studies to be analysed whilst maintaining a systematic approach. The thematic categorisation allows for concise summarisation and critical analysis of the complex hypoxic tumour lipidome.

Hypoxia elicits a wide range of adaptive lipidomic responses across the metabolic domains discussed, each representing potential opportunities for clinical translation. Broadly, the lipogenic phenotype established in hypoxia-adapted cancer cells confers a pro-tumorigenic survival advantage and innate protection within the harsh tumour microenvironment. Primarily driven by HIF signalling, lipid loading is achieved through coordinated induction of *de novo* lipogenesis utilising alternative carbon sources, increased exogenous lipid uptake and suppression of lipolysis. Once accumulated, lipids are reserved in lipid droplets and dynamically distributed to support tumour proliferation, rebalance the lipid desaturation index and minimise ER stress, preventing excessive lipid peroxidation. These tightly regulated processes depend on an extensive network of transcription factors, enzymes and proteins, many of which are potentially clinically actionable. Numerous studies demonstrated the prognostic value of monitoring levels of key lipid regulators including SREBP-1 and HILPDA [52, 59, 78]. Similarly, detecting elevated expression of hypoxia-associated lipid enzymes like FASN in tumour samples could signify inherent treatment resistance and prompt consideration of alternative therapeutic strategies [32]. However, given the invasive nature of tumour biopsies and the pronounced spatial and temporal heterogeneity of tumour hypoxia, non-invasive surrogates of the tumour lipidome may offer greater translational utility [102].

A further implication of hypoxia-driven lipid remodelling is altered susceptibility to ferroptosis, a regulated form of non-apoptotic cell death driven by iron-dependent lipid peroxidation. Ferroptosis sensitivity is strongly influenced by membrane phospholipid composition, particularly the abundance of peroxidation-prone polyunsaturated fatty acid-containing phospholipids, and by antioxidant systems such as glutathione peroxidase 4 (GPX4), which detoxify lipid hydroperoxides [103]. Several adaptations identified in this review, including altered desaturation balance, exogenous unsaturated lipid uptake, LD formation and sequestration of unsaturated lipids from ROS-mediated peroxidation, may therefore influence ferroptotic vulnerability in hypoxic tumours [18]. However, direct investigation of ferroptosis in physiologically hypoxic cancer models was not a major feature of the included literature. Defining how hypoxia shapes ferroptosis sensitivity through lipidomic remodelling represents an important future direction, particularly for treatment-resistant tumours [104].

**Fig. 6:**
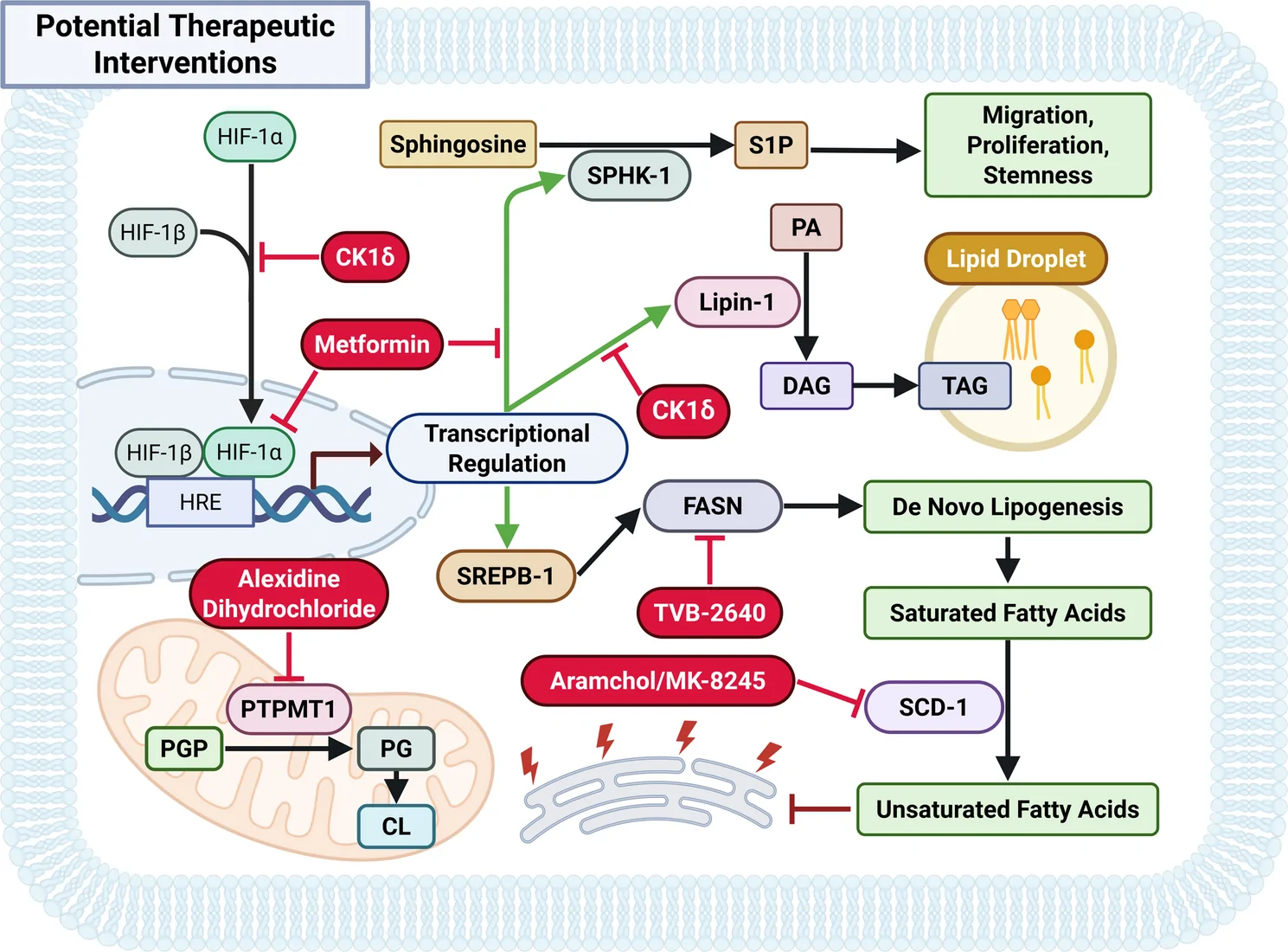
Potential Therapeutic Interventions: Potential therapeutic strategies include targeting HIF signalling, de novo lipogenesis, fatty acid desaturation, lipid droplet formation, sphingolipid signalling, and mitochondrial lipid metabolism. Agents such as CK1δ, metformin, TVB-2640, Aramchol/MK-8245, and alexidine dihydrochloride may disrupt HIF-driven transcription, FASN activity, SCD-1 function, Lipin-1-mediated TAG synthesis, SPHK-1/S1P signalling, and PTPMT1-dependent cardiolipin metabolism

Interrogating hypoxic lipid metabolism uncovers unique tumour vulnerabilities that may be therapeutically exploited (Fig. 6). The reliance of hypoxic cancer cells on exogenous unsaturated lipids is clinically actionable through novel therapeutics targeting CK1δ and FABP pathways [55, 56]. Similarly, SCD-1 inhibitors acting on acid-adapted tumour cells limit the pool of MUFAs available to neighbouring hypoxic cells, thereby compromising their survival [44]. Disrupting these pathways induces intolerable ER stress and activates UPR-mediated apoptosis. In parallel, advances in lipid signalling have identified new potential avenues for hypoxic-targeting medications, including the use of metformin and YME1L antagonists to disrupt oncogenic lipid signalling, as well as miR-15a-5p analogues to suppress tumour growth [69–71]. Additionally, the characterisation of hypoxia-induced membrane remodelling provides insights into both mechanisms of treatment resistance and emerging therapeutic vulnerabilities. On the one hand, hypoxic inhibition of FA2H reduces the clustering of lipid rafts and increases membrane rigidity, thereby impairing chemotherapeutic uptake, with important implications for treatment selection and efficacy [80]. On the other hand, hypoxia-driven membrane adaptations concurrently generate exploitable weaknesses. For example, disruption of mitochondrial membrane integrity using PTPMT1 inhibitors, such as alexidine dihydrochloride, can abrogate a critical protective mechanism that limits ROS accumulation [83].

These therapeutic approaches differ substantially in translational maturity. Metformin is already approved and widely used clinically as an anti-diabetic agent, although its role as an anti-cancer metabolic therapy remains investigational [70]. FASN inhibitors represent one of the more clinically advanced lipid-targeting strategies, with TVB-2640/denifanstat evaluated in early-phase oncology trials, including a first-in-human dose-escalation study and a phase II study in recurrent high-grade astrocytoma [105, 106]. In contrast, therapeutic strategies targeting SCD-1, CK1δ/FABP-dependent lipid uptake, YME1L-mediated lipid signalling, PTPMT1 inhibition and miR-15a-5p replacement remain largely preclinical in the specific context of hypoxia-driven lipid metabolic adaptation [44, 55, 56, 69, 71, 83]. Hypoxia-driven lipid metabolism may also intersect with immunotherapy response. Hypoxia can promote immune escape through HIF-dependent PD-L1 regulation and impaired T- and NK-cell effector function, while lipid accumulation, redox stress and altered ferroptosis sensitivity may influence immune-mediated tumour cell killing [5, 107–110]. However, immune endpoints such as PD-L1 expression, T-cell exhaustion and response to immune checkpoint blockade were rarely examined in the studies included in this review.

Dietary metabolic interventions, including high-fat and ketogenic diets, represent a related but distinct translational area. Experimental evidence suggests that systemic hyperlipidaemia combined with local tumour-cell lipid loading [111]; in a glioma model, high-fat diet-induced hyperlipidaemia augmented tumour vascularisation and macrophage infiltration and was associated with reduced survival [45]. However, the broader interactions between dietary interventions, tumour hypoxia and lipid metabolic adaptation remain incompletely characterised and were outside the predefined scope of this review, but merit further study.

From an experimental perspective, there are fundamental gaps in the existing literature. For example, there is a noticeable absence of publications in multiple cancer types, including oesophagogastric, head and neck and bladder cancers. There was also a tumour-lineage bias, with no included studies specifically investigating sarcoma models. This represents an important gap in understanding whether hypoxia-driven lipid metabolic adaptations differ in solid tumours of mesenchymal origin. As highlighted in this review, tumour responses to hypoxia are highly context-dependent due to cell-specific DNA methylation patterns and steric hindrance [112]. Therefore, systematic evaluation of the hypoxic lipidome across diverse cancer types is required, as findings from one tumour context may not be readily generalisable to others.

Selection of experimental oxygenation conditions represents a recurrent methodological limitation across the studies included in this review. Normoxic controls were frequently maintained at ambient oxygen levels of 20–21%, which are widely considered hyperoxic and unrepresentative of physiological tissue oxygenation [6]. Consequently, this may compromise the translational relevance and reliability of reported findings. Severe or radiobiological hypoxia (< 0.5%) was infrequently evaluated, despite its critical relevance to understanding therapeutic resistance in cancer [113]. Moreover, experimental models rarely incorporated acute or cycling hypoxia, although fluctuating oxygenation has been shown to promote genomic instability and mutagenic capacity, as well as resistance and metastatic potential [114].

In addition, there is a scarcity of data derived from human tumour tissue, with a heavy reliance on in vitro cell line models. Studies that examine hypoxia and lipid metabolism in human tissue primarily rely on immunohistochemical endogenous hypoxic markers, such as HIF and GLUT-1. However, these surrogate markers are often elevated in pseudohypoxic states and correlate poorly with true physiological tumour hypoxia [115]. Therefore, this field would be substantially advanced by clinical studies incorporating validated exogenous hypoxia markers, such as pimonidazole, to more accurately characterise hypoxia-driven lipid metabolic reprogramming in human cancers.

### Strengths and limitations

This is the first scoping review in the field, and this novelty strengthens its findings. The strict exclusion criteria for studies with hypoxic mimetics ensure findings represent lipidomic adaptations relevant to physiological hypoxia. No studies were excluded based on a quality or risk of bias assessment to avoid limiting the breadth of the review. However, this may have led to an overrepresentation of poor-quality articles. The review was restricted to the English language, which may have excluded some relevant studies.

## Conclusion

In conclusion, this scoping review provides a comprehensive overview of current knowledge on hypoxia-driven lipid metabolic reprogramming in solid tumours. The evidence highlights a complex adaptive response characterised by enhanced lipogenesis, lipid conservation, reliance on exogenous unsaturated lipids, and a central role for lipid droplets. These metabolic adaptations are further supported by intricate lipid signalling networks and remodelling of cellular and mitochondrial membranes. While the literature identifies several avenues for clinical translation, significant gaps remain in experimental design, model systems and the diversity of tumour types studied. Addressing these limitations through the use of more physiologically relevant models and expanded clinical investigation will be essential to translate insights on hypoxia-driven lipid metabolic changes into clinical benefit.

## Supplementary Information

Supplementary Material 1.

## Data Availability

No datasets were generated or analysed during the current study.

## Abbreviations

2D: two-dimensional
ACC: Acetyl-CoA Carboxylase
ACADM: Medium-Chain Acyl-CoA Dehydrogenase
ACADVL: Very long-Chain Acyl-CoA Dehydrogenase
ACSS2: Acetyl-CoA Synthetase 2
Akt: Protein Kinase B
ATP: Adenosine Triphosphate
ChKα: Choline Kinase-α
ccRCC: Clear Cell Renal Cell carcinoma
CK1δ: Casein Kinase 1 delta
CPT1: Carnitine Palmitoyltransferase-1
CPT1A: carnitine palmitoyltransferase 1 A
DNA: Deoxyribonucleic Acid
ER: Endoplasmic Reticulum
ETC: Electron Transport Chain
FABP: Fatty Acid Binding Proteins
FABP5: Fatty Acid Binding Protein 5
FA2H: Fatty Acid 2-Hydroxylase
FAO: Fatty Acid Oxidation
FASN: Fatty Acid Synthase
GDP1: Glycerol-3-Phosphate Dehydrogenase 1
GDP2: Glycerol-3-Phosphate Dehydrogenase 2
GLUT: 1-Glucose Transporter Type 1
HADHA: Hydroxyacyl-CoA Dehydrogenase trifunctional multienzyme complex subunit alpha
HC: Hypoxic Chamber
HCC: Hepatocellular Carcinoma
HIF: Hypoxia-inducible factor
HILPDA: Hypoxia-Inducible Lipid Droplet-associated
IDH: 1-Isocitrate Dehydrogenase-1
IDH: 2-isocitrate dehydrogenase-2
IRE: 1-Inositol-Requiring Enzyme-1
JNK: c-Jun N-terminal kinase
LCAD: Long-Chain Acyl-CoA Dehydrogenase
LD: Lipid Droplets
LDLR: Low-Density Lipoprotein Receptor
MCAD: Medium-Chain Acyl-CoA Dehydrogenase
MeSH: Medical Subject Headings
mTOR: Mammalian Target of Rapamycin
MUFA / MUFAs: monounsaturated fatty acid(s)
PDH: Pyruvate Dehydrogenase
PGC: 1α-Peroxisome Proliferator-activated Receptor Gamma Coactivator 1α
PHD3: Prolyl Hydroxylase Domain 3
PTPMT1: Protein-Tyrosine Phosphatase Mitochondrial 1
REDD: 1-Regulated in Development and DNA Damage-1
ROS: Reactive Oxygen Species
S1P: Sphingosine-1-Phosphate
SCD: 1-Stearoyl-CoA Desaturase-1
SFA: Saturated Fatty Acid
SOAT1: Sterol O-acyltransferase 1
SPHK: 1-Sphingosine Kinase-1
SREBP: 1-Sterol Regulatory Element-Binding Protein-1
TCA: Tricarboxylic Acid
TNBC: Triple Negative Breast Cancer
TSC1/TSC2: Tuberous Sclerosis Complex-1 and − 2
UPR: Unfolded Protein Response
VLDLR: Very-Low-Density Lipoprotein Receptor
