# Supplementary material for: The influence of hypoxia on cancer lipid metabolism: a scoping review
Source: Mol Cancer. 2026 Aug 5;25:263. doi: 10.1186/s12943-026-02758-8 (PMC13729564; doi:10.1186/s12943-026-02758-8)
Supplement: Supplementary file 1 [file 12943_2026_2758_MOESM1_ESM.docx]

| Set | Search Statement |
| --- | --- |
| 1 | exp Neoplasms/ |
| 2 | (cancer* or carcin* or malig* or tumour* or neoplas*).mp. [mp=ti, ab, tx, ct, hw, tc, id, ot, tm, mf, bt, tn, dm, dv, kf, fx, dq, cw, nm, ox, px, rx, ui, sy, ux, mx] |
| 3 | (lipid* or fatty acid*).mp. [mp=ti, ab, tx, ct, hw, tc, id, ot, tm, mf, bt, tn, dm, dv, kf, fx, dq, cw, nm, ox, px, rx, ui, sy, ux, mx] |
| 4 | exp Hypoxia/ |
| 5 | hypox*.mp. |
| 6 | 1 or 2 |
| 7 | 4 or 5 |
| 8 | 3 and 6 and 7 |

**Supplementary Table 1 – OVID Search Strategy**
